# Wirtschaftliche Implikationen der Corona-Krise und wirtschaftspolitische Maßnahmen

**DOI:** 10.1007/s10273-020-2628-0

**Published:** 2020-04-22

**Authors:** Peter Bofinger, Sebastian Dullien, Gabriel Felbermayr, Clemens Fuest, Michael Hüther, Jens Südekum, Beatrice Weder di Mauro

**Affiliations:** 1 Lehrstuhl für VWL u.a., Universität Würzburg, Sanderring 2, 97070 Würzburg, Deutschland; 2 Institut für Makroökonomie u. Konjunkturforschung, Hans-Böckler-Straße 39, 40476 Düsseldorf, Deutschland; 3 Institut für Weltwirtschaft, Kiellinie 66, 24105 Kiel, Deutschland; 4 ifo Institut, Poschingerstr. 5, 81679 München, Deutschland; 5 Institut der deutschen Wirtschaft Köln e.V., Konrad-Adenauer-Ufer 21, 50668 Köln, Deutschland; 6 Düsseldorfer Institut für Wettbewerbsökonomie, Heinrich-Heine-Universität Düsseldorf, Universitätsstraße 1, 40225 Düsseldorf, Deutschland; 7 CEPR Centre for Economic Policy Research, 2nd Floor, 33 Great Sutton Street, EC1V 0DX London, UK

**Keywords:** O40, I15, H12

## Abstract

Die Corona-Krise hat ihren Ausgangspunkt in China und große gesundheitliche sowie wirtschaftliche Schäden verursacht. Mittlerweile sind auch in Japan, Korea, Italien, Deutschland, Großbritannien, Frankreich, Spanien und vor allem den USA hohe und rapide wachsende Fallzahlen von Infektionen mit SARS-CoV-2 zu verzeichnen. Prognosen über das Wirtschaftswachstum mussten massiv nach unten revidiert werden, und weltweit ringen Regierungen mit der richtigen wirtschaftspolitischen Reaktion. Dieser Beitrag beschreibt grundsätzliche kurzfristige Handlungsoptionen für die deutsche Regierung auf den Corona-Schock zu reagieren und bewertet kurz den am 13. März 2020 von Bundesfinanzminister Olaf Scholz und Bundeswirtschaftsminister Peter Altmaier vorgestellten „Schutzschirm für Beschäftigte und Unternehmen“.

## Abstract (en)

The corona crisis started in China and had great consequences for public health and the economy. In the meantime, high and rapidly growing numbers of cases of infections with SARS-CoV-2 have also been recorded in Japan, Korea, Italy, Germany, Great Britain, France, Spain and above all in the USA. Forecasts of economic growth have been massively revised downwards and governments around the world are struggling to find the right economic policy response. This article describes basic short-term options for the German government to react to the corona shock and briefl y assesses the package of measures “Schutzschirm für Beschäftigte und Unternehmen” presented on 13 March 2020 by the German Finance Minister Olaf Scholz and the German Economics Minister Peter Altmaier.

Die wesentliche Schwierigkeit im wirtschaftspolitischen Umgang mit dem Coronavirus ergibt sich daraus, dass es in den betroffenen Volkswirtschaften sowohl einen Angebots- als auch einen Nachfrageschock auslöst.[1] Der massive Einbruch an den Börsen und die Flucht in sichere Anlagen ist eine Reaktion auf diese Schocks, kann aber weitere Erschütterungen auslösen und die Abwärtsdynamik in der Realwirtschaft verstärken. Das Ausmaß dieser Schocks ist stark von Erwartungen und damit von psychologischen Faktoren abhängig. Wirtschaftspolitische Maßnahmen müssen auf diese Spezifika hin abgestimmt und zielgerichtet sein. Entscheidende Bedeutung haben dabei das Timing und die Kommunikation der Maßnahmen. Das wichtigste Ziel besteht in der Sicherung des Vertrauens, dass die Gesundheitskrise nicht zu einer systemischen Wirtschaftskrise wird, die den Arbeitsmarkt, die Banken und Finanzmärkte erfasst und so die heimische Nachfrage zusätzlich schwächt.

## Das Coronavirus als Angebotsschock

*Globale Wertschöpfungsketten*: Bei weltweit hoch integrierten Wertschöpfungsketten übertragen sich Produktionsausfälle aufgrund fehlender Vorleistungen auch auf Unternehmen außerhalb der Epidemiezentren. Werden essenzielle Vorprodukte aus China oder dem industrialisierten Norden Italiens nicht geliefert, können auch in Deutschland Produktionsprozesse zum Stillstand kommen. Problematisch ist hierbei insbesondere, dass viele Unternehmen heute auf eine Just-in-time-Produktion mit niedrigen Lagerbeständen setzen. Außerdem sind die zugelieferten Komponenten oftmals extrem spezialisiert und auf die Spezifika des jeweils nächsten Schritts in der Wertschöpfungskette zugeschnitten. Sie werden häufig nur in einem oder wenigen Zulieferbetrieben hergestellt. Es gibt also für deutsche Betriebe, die auf chinesische Vorprodukte warten, oftmals weltweit keine Alternativanbieter, die in vertretbarer Zeit und zu akzeptablen Preisen entsprechende Komponenten liefern könnten.

Vieles spricht dafür, dass der deutschen Volkswirtschaft der Höhepunkt dieser Produktionsausfälle noch bevorsteht. Die Betriebsschließungen in China erreichten Anfang Februar 2020 ihren Höhepunkt, mittlerweile läuft die Produktion langsam wieder an. Auch aufgrund der zeitlichen Verzögerungen auf dem Transportweg (sechs Wochen auf dem Seeweg) wird das Ausmaß des wirtschaftlichen Schadens in Deutschland erst nach und nach offenbar. Der Schaden wird umso gravierender, je weniger es den Betrieben gelingt, betriebsspezifische Lösungen zu finden und die Lücke durch Alternativangebote oder eigene Produktionen zu schließen. In dem Maß, in dem sich das Virus in Europa ausbreitet und umfassende Quarantänemaßnahmen in den Ländern ergriffen werden, kommt es zu Störungen in weiteren Lieferketten.

*Ausfall von Beschäftigten*: Ein weiterer Angebotsschock ergibt sich, wenn Mitarbeiter ausfallen und für die Produktion nicht mehr zur Verfügung stehen. Dies kann direkt aufgrund eigener Erkrankung oder durch die Verhängung

1 Die aktuelle Lage bezüglich der Corona-Krise verändert sich rasend. Der vorliegende Text wird deshalb in Teilen zum Zeitpunkt der Veröffentlichung von der Realität überholt sein. Die Analyse basiert auf der von den Autoren am 11. März 2020 auf der Bundespressekonferenz vorgestellten Kurzstudie „Wirtschaftliche Implikationen der Corona-Krise und wirtschaftspolitische Maßnahmen".

**Prof. Dr. Peter Bofinger** war Lehrstuhlinhaber für VWL I, Geld und internationale Wirtschaftsbeziehungen an der Universität Würzburg.

**Prof. Dr. Sebastian Dullien** lehrt Volkswirtschaftslehre an der Hochschule für Technik und Wirtschaft Berlin und ist Wissenschaftlicher Direktor des Instituts für Makroökonomie und Konjunkturforschung in der Hans-Böckler-Stiftung (IMK) in Düsseldorf.

**Prof. Gabriel Felbermayr**, Ph.D., ist Präsident des Instituts für Weltwirtschaft (IfW) in Kiel.

**Prof. Dr. Dr. h.c. Clemens Fuest** ist Präsident des ifo Instituts und Professor für Volkswirtschaftslehre, Lehrstuhl für Nationalökonomie und Finanzwissenschaft, an der Ludwig-Maximilians-Universität München.

**Prof. Dr. Michael Hüther** ist Direktor des Instituts der deutschen Wirtschaft (IW) und Hochschullehrer an der European Business School in Oestrich-Winkel.

**Prof. Dr. Jens Südekum** ist Professor am Düsseldorf Institute for Competition Economics (DICE) der Heinrich-Heine-Universität Düsseldorf.

**Prof. Dr. Beatrice Weder di Mauro** ist Präsidentin des Centre for Economic Policy Research in London.

einer Quarantäne geschehen. Auch die Schließung von Schulen und vorschulischen Einrichtungen kann indirekt zum Ausfall von Arbeitskräften führen, da sie nun in der heimischen Kinderbetreuung gebunden sind. Ähnlich verhält es sich möglicherweise bei Erkrankungen im familiären Umfeld, wenn die Krankenbetreuung nicht durch das Gesundheitssystem sichergestellt werden kann. Eine Abmilderung dieses Angebotsschocks kann prinzipiell durch Varianten der Tele- und Heimarbeit geschaffen werden. Diese Optionen werden ohnehin in vielen Wirtschaftszweigen immer beliebter, sie stehen aber längst nicht allen Beschäftigten gleichermaßen zur Verfügung. Insbesondere bei den personenbezogenen Dienstleistungen oder bei Produktionsberufen mit hohem manuellen Routineanteil ist diese Option keine realistische Ausweichreaktion.

*Störung der Infrastrukturen und Logistikketten*: Ein besonderes Problem zur Aufrechterhaltung der Produktionsketten kann sich durch die Beeinträchtigung der Verkehrsinfrastrukturnetze ergeben, wenn über längere Zeit Flugverbindungen stark ausgedünnt oder storniert werden und wenn der Bahnbetrieb beeinträchtigt ist, weil Zugverbindungen wegen Evakuierungen oder fehlendem Personal nicht mehr verlässlich funktionieren. In China zeigt sich, dass selbst nach Wiederaufnahme der Arbeit und damit der Produktion die Logistikketten noch länger nicht verlässlich sind, weil viele Transportmittel an der verkehrten Stelle stehen und größere Warenbestände den reibungslosen Ablauf nicht sogleich möglich machen. Deshalb ist selbst nach einem Ende der Quarantäne ein längerer Nachlauf der Störungen zu erwarten.

## Das Coronavirus als Nachfrageschock

*Globale Nachfrage nach deutschen Gütern*: China und andere stark vom Coronavirus betroffene Volkswirtschaften werden auf absehbare Zeit ihre Nachfrage nach deutschen Vorprodukten, Investitions- und Konsumgütern sowie nach Tourismus-Dienstleistungen reduzieren. Das volle Ausmaß dieser globalen Auswirkungen ist noch nicht absehbar. Es hängt insbesondere vom weiteren Verlauf der globalen Verbreitung des Virus ab. Die OECD hat hierzu aber erste modellgestützte Abschätzungen und Prognosen vorgelegt (Boone, 2020). Das sehr optimistischen Szenario, bei dem die Virusverbreitung im Wesentlichen auf China konzentriert bleibt und das globale Wachstum des Bruttoinlandsprodukts (BIP) durch das Coronavirus im Jahr 2020 um etwa 0,5 Prozentpunkte zurückgeht (von 2,9 % auf 2,4 %), ist inzwischen überholt, und das pessimistische Szenario, in dem sich das Virus auch außerhalb Chinas rapide verbreitet, ist längst Realität geworden. Wirtschaftliche Auswirkungen ergeben sich dann nicht bloß durch den Rückgang der chinesischen Nachfrage, sondern auch direkt in den betroffenen Ländern. Im pessimistischen Szenario der Organisation für wirtschaftliche Zusammenarbeit und Entwicklung (OECD) sinkt das globale BIP-Wachstum im Jahr 2020 um 1,5 Prozentpunkte und das Welthandelsvolumen um 3,75 %. In anderen vorgelegten Prognosen ergeben sich sogar noch weitaus dramatischere Auswirkungen.[2] Deutschland wäre aufgrund seiner Wirtschaftsstruktur (Offenheitsquote und Industrieanteil) von diesen Entwicklungen besonders betroffen. So gehen auch McKibbin und Fernando (2020) davon aus, dass Deutschland in den verschiedenen von ihnen betrachteten Szenarien innerhalb Europas überproportional stark von der Corona-Krise betroffen ist.

*Heimische Nachfrageschocks I – Tourismus und „sozialer Konsum“*: Negative Auswirkungen auf der Nachfragesei-

2 Nach McKibbin, und Fernando (2020) können sich die Auswirkungen des Coronavirus auf das globale BIP im Jahr 2020 im pessimistischsten Fall auf bis zu -7 % belaufen.

te ergeben sich auch direkt aufgrund binnenwirtschaftlicher Prozesse, indem der „soziale Konsum“ massiv eingeschränkt wird. Hierunter sind etwa Restaurantbesuche, Inlandstourismus, der Besuch von Kulturveranstaltungen, Messen usw. zu verstehen (Wren-Lewis, 2020). Nachdem frühzeitig eine Reihe prominenter Veranstaltungen (wie die Hannover-Messe oder die Leipziger Buchmesse) abgesagt worden war, haben mittlerweile alle Sportbundesligen den Betrieb eingestellt. Deutschlandweit wurden Kultureinrichtungen, Kneipen und Clubs geschlossen. Eine besondere Problematik für diesen Nachfrageausfall besteht darin, dass nicht zwingend mit zeitlichen Nachholeffekten zu rechnen ist. Bei unterbrochenen Lieferketten kann in vielen Fällen damit gerechnet werden, dass die entsprechenden Käufe und Verkäufe später (gegebenenfalls noch im Verlauf des Jahres 2020) erfolgen. Wenn ein Auto heute nicht produziert und verkauft werden kann, dann vielleicht in ein paar Monaten. Dies ist beim sozialen Konsum anders. Heute nicht stattfindende Restaurantbesuche oder Privatreisen werden nicht unbedingt später nachgeholt. Der private Verbrauch hat sich bisher als Konjunkturstütze erwiesen. Aufgrund der Stornierung von Messen, Reisen und Veranstaltungen ist eine Konsum-Rezession im 1. Halbjahr 2020 zusätzlich zur ohnehin schon einige Quartale laufenden Industrierezession sehr wahrscheinlich geworden. Ob eine vollständige Kompensation dieser Effekte in den Folgequartalen realistisch erscheint, ist zumindest fraglich, weil bestimmte Nachfragekomponenten nicht unbedingt nachgeholt werden.

*Heimische Nachfrageschocks II – Verunsicherung und Vorsichtssparen*: Neben dem „sozialen Konsum“ können weitere Nachfragesegmente negativ von der Corona-Krise betroffen sein. So mag die Aussicht auf eine längere Quarantäne zwar bei bestimmten Gütern zu Vorrats- und Hamsterkäufen führen, verunsicherte Konsumenten könnten dafür aber vor größeren Anschaffungen oder dem Kauf langlebiger Konsumgüter bis hin zum Bau neuer Eigenheime zurückschrecken. Dies gilt besonders für solche Konsumenten, die permanente Einkommensverluste durch die Corona-Krise befürchten. Dies wäre z. B. bei einem drohenden Arbeitsplatzverlust der Fall. Denn die bisherige Konsumplanung bezog sich auf den Einkommensstrom des bisherigen Beschäftigungsverhältnisses. Wenn der Arbeitgeber aber aufgrund Corona-bedingter Liquiditätsprobleme nun zu Entlassungen greifen muss, bzw. wenn diese von den Beschäftigten erwartet werden, dann kann dies zu einem erhöhten Vorsichtssparen und damit zur Anpassung der bisherigen Konsumpläne führen. Mögliche Nachholeffekte wären für diese Personengruppe erst dann zu erwarten, wenn die wirtschaftlichen Belastungen des Corona-Schocks vollständig wieder abgeklungen sind und nicht zu lang andauernden Hysteresis-Effekten geführt haben, die im Fall von unfreiwilligen und ungeplanten Entlassungen aber oftmals auftreten.

## Unterschied zur Finanzkrise

In der Finanzkrise entlud sich ein über Jahre hinweg aufgebautes Ungleichgewicht auf den Immobilienmärkten, mit erheblichen negativen Effekten auf das Finanzsystem. Verschärft wurde die Krise durch einen Kollaps des Vertrauens der Banken untereinander. Der damit verbundene Verfall der Werte von Finanzaktiva und Immobilien hatte erhebliche Vermögenseffekte. Hinzu kam eine Kontraktion der Kreditvergabe. All dies hat zu einem Einbruch sowohl der Konsumausgaben als auch der privaten Investitionen geführt. Der Abbau der Überschuldung von Firmen und privaten Haushalten hat die Erholung der Wirtschaft verzögert. Ein Angebotsschock lag im Kontext der Finanzkrise aber nicht vor. Daher konnte die Politik sich auf die Stabilisierung des Finanzsystems und der Konsum- und Investitionsnachfrage konzentrieren.

Bei Corona ist die Lage insofern komplexer, als ein simultaner Angebots- und Nachfrageschock vorliegt, und der Einbruch der Nachfrage nach „sozialem Konsum“ aus medizinischen Gründen nicht durch staatliche Nachfragestützung ausgeglichen werden soll und wohl auch nicht kann. Dort, wo Angebotsausfälle auftreten, ist eine staatliche Nachfragestützung eher kontraproduktiv; hier geht es um staatliche Maßnahmen, die Störungen auf der Angebotsseite entgegenwirken. Die Aussichten auf wirtschaftliche Erholung sind insofern besser, als nach Ende der Epidemie zügiger wieder Normalbetrieb aufgenommen werden könnte und positive Nachholeffekte die Erholung unterstützen würden. Das setzt allerdings voraus, dass die Epidemie unter Kontrolle gebracht wird und negative Wirkungen mit andauernder Wirkung, wie eine Insolvenzwelle und vor allem größere Entlassungswellen, ausbleiben. Eine weitere Parallele besteht darin, dass es auch in der Corona-Krise wichtig ist, einem Verfall des Vertrauens unter Unternehmen der Realwirtschaft und im Finanzsektor in die Solidität und Zahlungsfähigkeit der Geschäftspartner entgegenzuwirken. Dieser würde nämlich, ähnlich wie 2008, zu einer Run-Situation, also einer durch fundamentale Faktoren nicht begründeten Panik führen.

## Medizinische Versorgung als oberste Priorität

Die oberste Priorität bei allen (wirtschafts-)politischen Maßnahmen muss darin bestehen, die Funktionalität des Gesundheitssystems und der medizinischen Versorgung sicherzustellen. Eine dysfunktionale medizinische Versorgung – also überfüllte oder gar geschlossene Arztpraxen und Kliniken, überforderte Gesundheitsämter, fehlende Medikamente und Testmöglichkeiten – würden maßgeblich zum Entstehen einer Überreaktion in der Bevölkerung beitragen, zumal wenn sie von einem weiteren Anstieg der Infektionsfälle begleitet wird. Das Entstehen einer solchen Dynamik gilt es unter allen Umständen zu vermeiden, auch wenn dafür ein gezielter Verzicht auf „sozialen Konsum" (wie z. B. die Absage von Großveranstaltungen) sinnvoll und konsequent geboten ist. Statisch betrachtet gibt es zunächst einen Konflikt zwischen medizinischen und ökonomischen Zielen. Die Absage einer Veranstaltung oder das Schließen von Schulen verlangsamt einerseits die Verbreitung des Virus.[3] Andererseits rufen diese Maßnahmen unmittelbare Kosten hervor, z. B. weil Beschäftigte nicht arbeiten dürfen oder in der Kinderbetreuung gebunden sind. Langfristig wären die ökonomischen Auswirkungen eines zu laschen Ansatzes bei der Eindämmung des Coronavirus jedoch deutlich schlimmer. Denn wenn sich der Infektionsverlauf exponentiell fortentwickelt und das Gesundheitssystem an seine Kapazitätsgrenzen stößt, drohen in der Zukunft viel drastischere Quarantäne-Maßnahmen und damit auch höhere Kosten.

Eine staatliche Garantie der Gesundheitsversorgung ist voraussetzungsreich, weil reale Ressourcenbeschränkungen (z. B. Verfügbarkeit von medizinischem Personal und Medikamenten) eine gewichtige Rolle spielen. Im Rahmen seiner Möglichkeiten sollte der Staat aber alles tun, um diese Funktionalität so gut wie nur irgend möglich aufrechtzuerhalten. Hierfür sollten einerseits die nötigen Finanzmittel mit höchster Priorität garantiert werden. Weitere, auch unkonventionelle, Maßnahmen sind zu erwägen, wenn es zu angebotsseitigen Engpässen kommt. Zum Beispiel:

- Im Ruhestand befindliches medizinischen Personal sollte reaktiviert (wie z. B. in Großbritannien), Urlaubssperren ausgesprochen und Dienstreisen untersagt werden.
- Finanzielle Anreize für niedergelassene Ärzte in Teilzeit, die Arbeitszeit vorübergehend auszudehnen, sollten geschaffen werden – insbesondere, wenn andere Praxen wegen Infektionsfällen geschlossen werden.
- Im Fall der Schließung von Schulen und vorschulischen Einrichtungen sollten spezielle Betreuungsangebote für die Kinder des medizinischen Personals gemacht werden, damit dieses seiner Arbeit nachgehen kann.
- Der Staat kann durch entsprechende Nachfrage Firmen – zumal solche, deren Produktion ohnehin stillsteht – dazu bewegen, im Rahmen ihrer Möglichkeiten die Produktpalette auf dringend benötigte medizinische Güter umzustellen (z. B. Atemschutzmasken).
- Beschäftige im öffentlichen Dienst, die bedingt durch das Coronavirus ihrer normalen Tätigkeit nicht nachgehen, können ersatzweise den Gesundheitssektor unterstützen (z. B. die Gesundheitsämter bei der Aufrechterhaltung von Bürgertelefonen etc.).

3 Die Effektivität gezielter Eindämmungsmaßnahmen scheint dabei recht hoch zu sein. Vgl. World Health Organization, 2020.

## Wirtschaftspolitische Maßnahmen

Bei einer nur temporären Störung im Fadenkreuz von gleichzeitigen Angebots- und Nachfrageschocks geht es überwiegend darum, negative Effekte auf die Liquidität der Unternehmen und damit auch auf die Stabilität des Bankensystems zu kompensieren. Bei einer länger anhaltenden Störung muss es zunehmend darum gehen, zusätzlich die Ertragslage der Unternehmen zu stabilisieren und negative Effekte auf den Arbeitsmarkt zu vermeiden. Es ist wichtig zu erkennen, dass Forderungen nach einem Konjunkturpaket, d. h. den üblichen Maßnahmen der Konjunkturstimulierung durch Geld- und Fiskalpolitik, den Besonderheiten dieser Krise nicht gerecht werden. Drei Aspekte sind zentral:

1. Es geht darum, gezielt wirtschaftliche Aktivitäten zu stimulieren, die nicht zu einer Ausbreitung der Pandemie führen. „Sozialer Konsum", der Menschen physisch zusammenbringt, sollte deshalb nicht stimuliert werden. Demgegenüber sollten Handel und Kapitalströme soweit wie möglich stabilisiert werden.

2. Die Zeitverzögerung bei einem klassischen Investitionsprogramm wäre viel zu groß, um einen wirksamen Beitrag zur Eindämmung der unmittelbaren wirtschaftlichen Folgen der Corona-Krise zu leisten.

3. Es besteht die Gefahr, dass sich selbstverstärkende pessimistische Erwartungen zu einer konjunkturellen Abwärtsspirale führen. Wenn Unternehmen wegen der Krise ein deutlich erhöhtes Risiko sehen, dass ihre Geschäftspartner das Vertrauen von Kunden, Lieferanten oder Kapitalgebern verlieren, kann es zum Abbruch von Geschäftsbeziehungen und einer Welle von Unternehmensinsolvenzen kommen. Hier können staatliche Maßnahmen wie Liquiditätsprogramme, temporäre steuerliche Hilfen oder Kurzarbeitergeld nicht nur den direkt betroffenen Unternehmen helfen, sondern die Erwartungen unmittelbar stabilisieren.

## Beitrag der Europäischen Zentralbank ist begrenzt

Für den Fall, dass es zu einer allgemeinen Vertrauenskrise in die Stabilität des Finanzsystems und damit zu einem „Bank Run" käme, bei dem Einleger ihre Einlagen von Banken abziehen, muss die Europäische Zentralbank (EZB) als „Lender of Last Resort" uneingeschränkt bereitstehen. Dies hat EZB-Präsidentin Christine Lagarde auch bereits auf der Pressekonferenz am 12. März 2020 angekündigt. Für Liquiditätsengpässe von Banken verfügt die EZB über die erforderlichen Refinanzierungsinstrumente (z. B. die langfristige Geldbereitstellung – LTRO). Die Spielräume der EZB zur Beeinflussung der Zinsbelastung von Unternehmen sind jedoch äußerst begrenzt. Dies gilt für weitere Zinssenkungen am kurzen wie am langen Ende im Rahmen der konventionellen Geldpolitik sowie im Rahmen von Anleihekäufen.

Der Ankauf von Unternehmensanleihen durch die EZB, z. B. von Fluggesellschaften, wäre möglich. Es ist aber ordnungspolitisch problematisch, weil viel kleine und mittlere Unternehmen ebenfalls von der Krise betroffen sind, davon aber nicht profitieren würden. Der Ankauf von Aktien, wie er durch die japanische Notenbank betrieben wird, ist ebenfalls kritisch zu sehen. Bisherige Erfahrungen mit Kurseinbrüchen an den Börsen zeigen, dass die davon ausgehenden Effekte für die Gesamtwirtschaft begrenzt sind. Die Lösung des Helikoptergelds wäre schon technisch nur schwer umzusetzen, ganz abgesehen von der fehlenden Zielgerichtetheit.

### Fiskalpolitik verfügt über größere Spielräume für die Stabilisierung auf kurze und mittlere Sicht

Grundsätzlich sollte für alle fiskalpolitischen Maßnahmen das Prinzip: timely, targeted, temporary gelten (Gaspar und Mauro, 2020). Die Corona-Krise ist also für sich genommen kein Anlass für dauerhafte Steuersenkungen oder eine permanente Ausweitung der Staatsausgaben. Die Spielräume für die Fiskalpolitik werden in einer derartigen Krisensituation nicht durch die Schuldenbremse eingeschränkt. Die Schuldenbremse weist explizit eine Ausnahme für Krisensituationen (Naturkatastrophen, außergewöhnliche Ereignisse außerhalb der Kontrolle des Staates) auf (Artikel 115 Grundgesetz); der Stabilitäts- und Wachstumspakt sieht in Krisensituationen (außergewöhnliche Ereignisse, außerhalb der Kontrolle der Mitgliedstaaten) ebenfalls temporäre fiskalische Spielräume vor. Im Gegensatz zur EZB verfügt die deutsche Finanzpolitik, nicht zuletzt wegen der im internationalen Vergleich niedrigen Schuldenstandquote, über große Potenziale zur Stabilisierung der Wirtschaft auf kurze wie auch mittlere Sicht. Ein Festhalten am haushaltspolitischen Ziel der schwarzen Null würde dazu führen, dass diese Spielräume nicht genutzt werden könnten.

### Liquiditätshilfen

Auf kurze Sicht führt der Nachfrageschock für viele Unternehmen zu einem Liquiditätsproblem. Während Einzahlungen ausblieben, müssen Zahlungen für laufende Verpflichtungen (insbesondere für Löhne, Mieten, Zins- und Tilgungsverpflichtungen, Pachten und Leasing-Verpflichtungen etc.) weiterhin geleistet werden. Es besteht somit die Gefahr, dass es – zunächst bei schwächeren Unternehmen – zu Insolvenzen kommt. Mit dem Verlauf der Krise könnten dann aber auch liquidere Unternehmen erfasst werden. Insbesondere besteht die Gefahr, dass es durch in bestimmten Sektoren gehäufte Insolvenzen zu unerwünschter Marktkonzentration kommt. Es muss also wirtschaftspolitisch darum gehen, durch umfassende Liquiditätshilfen möglichst rasch und unbürokratisch dafür zu sorgen, dass die Corona-Krise nicht zu einer Insolvenzwelle für die deutsche Wirtschaft führt. Dem hat die Bundesregierung mit dem Maßnahmenpaket „Ein Schutzschild für Beschäftigte und Unternehmen“ vom 13. März 2020 Rechnung getragen (Bundesministerium der Finanzen, 2020).

- Traditionell werden Liquiditätshilfen durch die Kreditanstalt für Wiederaufbau (KfW) vergeben. Sie verfügt hierfür über das KfW-Mittelstandsprogramm mit einem Jahresumsatz von bis zu 500 Mio. Euro. Unternehmen erhalten dabei langfristige, zinsgünstige Darlehen, die über Banken oder Sparkassen durchgeleitet werden. Die Bundesregierung hat am 13. März 2020 richtigerweise angekündigt, die entsprechenden Programme massiv auszuweiten, die Umsatzgrenzen deutlich anzuheben, die Zweckbindung zu lockern und die Risikoübernahme durch den Bund zu erhöhen. Dazu soll der Garantierahmen im Bundeshaushalt um 93 Mrd. Euro erweitert werden.

- Ein Beitrag zur Verbesserung der Liquidität von Unternehmen kann auch über großzügigere Abschreibungserleichterungen erreicht werden. Allerdings ist eine solche Maßnahme nur für Unternehmen hilfreich, die trotz der Krise zu Investitionen bereit und auch in der Lage sind.

- Ähnlich zu beurteilen ist eine großzügigere Gewährung des Investitionsabzugsbetrags: Dieser mindert den laufenden steuerlichen Gewinn, indem 40 % der voraussichtlichen Anschaffungskosten von in den nächsten drei Jahren geplanten Investitionen angesetzt werden können. Der Unterschied zu einer klassischen Sonderabschreibung besteht darin, dass keine Ausgaben in diesem Jahr erfolgen müssen. Der Betrag ist auf 200.000 Euro im Jahr und auf Unternehmen mit einem Betriebsvermögen bis zu 235.000 Euro beschränkt. Eine temporäre deutliche Anhebung beider Grenzen würde einen spürbaren Steuerstundungseffekt bewirken.

- Als administrativ sehr einfach und zugleich schnell wirkende Maßnahme wäre an eine generelle zinslose Stundung fälliger Steuerzahlungen (Vorauszahlungen und Nachzahlungen) der Einkommensteuer, Körperschaftsteuer und der Umsatzsteuer zu denken. Die Stundung sollte so lange bestehen, bis die Corona-Krise definitiv überwunden ist. Aus der Sicht des Budgets hätte diese Maßnahme den Vorteil, dass sie sich nicht nachteilig auf den Saldo auswirkt, solange die Stundung sich innerhalb des laufenden Kalenderjahrs bewegt. Hier hat die Bundesregierung am 13. März 2020 angekündigt, Stundungen zu erleichtern und Vorauszahlungen leichter anzupassen.

### Solvenzhilfen in der Corona-Krise

Schon jetzt zeigt sich, dass die Corona-Krise über reine Liquiditätseffekte hinaus mit negativen Effekten für die Ertragslage von Unternehmen verbunden ist. Da diese zunehmend dadurch verursacht werden, dass die Gesundheit der Bevölkerung als ein öffentliches Gut gesichert werden soll, ist es naheliegend, dass die damit einhergehenden ökonomischen Einbußen von der Gemeinschaft zumindest anteilig mitgetragen werden müssen. Ein klar sinnvolles und zielführendes Instrument ist, die Voraussetzungen für den Bezug von Kurzarbeitergeld zu erleichtern und den Unternehmen die Kosten der Sozialversicherungsbeiträge, die normalerweise auch im Falle von Kurzarbeit noch gezahlt werden müssen, zum Teil oder sogar vollständig zu erstatten. Die Beschlüsse des Koalitionsausschusses vom 8. März 2020 wurden mittlerweile gesetzkräftig. Seit dem 1. April gilt:

- Das Quorum der im Betrieb Beschäftigten, die vom Arbeitsausfall betroffen sein müssen, um Kurzarbeitergeld zu beantragen, wurde auf 10 % abgesenkt.
- Die Verpflichtung, zunächst im Rahmen der internen Flexibilität negative Arbeitszeitsalden aufzubauen, wurde ganz oder teilweise ausgesetzt.
- Kurzarbeitergeld ist auch für Leiharbeiter zu beanspruchen.
- Die Sozialbeiträge werden vollständig von der Bundesagentur für Arbeit erstattet.

Es geht bei Solvenzhilfen aber nicht bloß darum, Unternehmen die Weiterbeschäftigung ihrer Mitarbeiter zu ermöglichen. Vielmehr bedarf es zusätzlicher Unterstützung, um die negativen Effekte auf die Ertragslage abzumildern. Hierfür sind vor allem steuerpolitische Maßnahmen mit temporärer Wirkung erforderlich. Zu denken wäre hierbei prinzipiell an die durch das Stabilitäts- und Wachstumsgesetz geschaffene Möglichkeit einer temporären Herabsetzung der Einkommen- und Körperschaftsteuer (§ 51 Abs. 3 EStG und § 23 Abs. 2 KStG). Dabei stellt sich jedoch das Problem, dass Unternehmen, die in diesem Jahr aufgrund der Krise keine Gewinne erzielen, nicht in den Vorteil einer solchen Begünstigung kämen. Zielführender wären deshalb steuerliche Maßnahmen, die auch den Unternehmen zugute kommen, die in diesem Jahr größere Verluste erleiden. Das Mittel der Wahl hierfür ist der steuerliche Verlustrücktrag, der durch § 10d EStG vorgesehen ist und für Einkommen- und Körperschaftsteuer gleichermaßen gilt. In Anbetracht des Ausmaßes der Krise stellt sich bei dieser Regelung das Problem, dass der Verlustrücktrag bisher auf 1 Mio. Euro begrenzt ist. Eine Ausweitung dieser Grenze würde es mittleren und großen Unternehmen ermöglichen, in angemessener Weise an dieser Maßnahme zu partizipieren.

Im Vergleich dazu stellt das Vorziehen der Teilabschaffung des Solidaritätszuschlags auf den 1. Juli 2020 eine sehr viel begrenztere Maßnahme dar, die jedoch (allein aus psychologischen Gründen) zu begrüßen wäre. Sie erhöht unmittelbar die verfügbaren Einkommen weiter Teile der Bevölkerung. Dies kann zu relativ geringen fiskalischen Kosten das Vertrauen in die Handlungsfähigkeit der Politik und in eine rasche wirtschaftliche Belebung nach dem Abflauen der Corona-Krise stärken. Zur Stimulierung der Wirtschaft könnte auch an eine Senkung der Mehrwertsteuersätze gedacht werden. Diese Maßnahme würde einer allgemeinen Stimulierung der Konsumnachfrage dienen. Sie ist jedoch wenig zielgenau, da sie auch Bereichen zugute käme, die wie die Bauwirtschaft oder die Gebrauchsgüterindustrie nur begrenzt von der Krise betroffen sind. Auch ist unklar, inwieweit eine Steuersenkung überhaupt an die Konsumenten weitergegeben würde und damit Mehrkonsum anregen würde. Dafür erscheinen die fiskalischen Kosten (rund 10 Mrd. Euro Steuermindereinnahmen pro Umsatzsteuerpunkt) unverhältnismäßig hoch.

### Unternehmensrettungsfonds als Ultima Ratio?

Wenn es nicht gelingen sollte, die Ausbreitung der wirtschaftlichen Schockwellen einzudämmen, und es in größerem Umfang zu Unternehmensinsolvenzen käme, wäre als Ultima Ratio an Maßnahmen zu denken, bei denen sich der Staat mit Eigenkapital an Unternehmen beteiligt. Dies wäre analog zur Rettung von Banken im Rahmen des Banken-Schutzschirms SoFFin (Sonderfonds Finanzmarktstabilisierung) aus der Krise 2008/2009. Gerade für Großunternehmen wäre eine solche Lösung verhältnismäßig einfach umzusetzen. Im Vergleich zum Bankensystem gibt es in der Realwirtschaft jedoch eine weitaus größere Zahl von kleinen und mittleren Unternehmen, sodass die Umsetzung einer solchen Maßnahme mit einem enormen administrativen Aufwand verbunden wäre. Auch unter wettbewerbspolitischen Gesichtspunkten erscheint eine solche Lösung nicht unproblematisch.

### Herausforderungen für die Eurozone und die EU

Die Corona-Krise stellt auch die Eurozone vor große Herausforderungen. Mit Italien ist ein Land massiv getroffen, das ohnehin eine gefährlich hohe Staatsverschuldung hat und für das bereits in der Vergangenheit die Risikoprämien in Zeiten fiskalischer Belastung oder politischer Unsicherheit gefährlich in die Höhe geschossen sind. In der aktuellen Situation muss verhindert werden, dass sich aus den zusätzlichen fiskalischen Belastungen aus der Corona-Krise eine Situation ergibt, in der Investoren die Risikoprämien auf italienische Bonds so weit in die Höhe treiben. Hierzu müssen die Euro-Partner bereit sein, schnell ein Programm des Europäischen Stabilitätsmechanismus (ESM) auf den Weg zu bringen, wobei die Kondinitionalität so gestaltet werden sollte, dass das Programm für die italienische Regierung akzeptabel ist und keine zusätzliche kurzfristige Belastung für die italienische Wirtschaft bedeutet. Solch ein ESM-Programm würde auch den Weg für eine Nutzung der Outright Monetary Transactions (OMT) durch die EZB freimachen, was alleine die Erwartungen an den Finanzmärkten stabilisieren dürfte.

Eine andere Gefahr stellen Grenzschließungen und Exportbeschränkungen innerhalb der EU dar, die Versorgungsprobleme mit wichtigen (medizinischen) Gütern und neuen wirtschaftlichen Schaden durch Unterbrechung von Lieferketten verursachen könnten, insbesondere, da nicht alle Länder in der EU heute noch zentrale medizinische Ausrüstung wie etwa Beatmungsgeräte produzieren. So sollten die Grenzen für Waren unbedingt offen gehalten werden. Exportbeschränkungen sollten eine absolute Ultima Ratio sein, die nur eingesetzt wird, wenn die Versorgung der eigenen Bevölkerung mit vitalen medizinischen Gütern nicht anders sicherzustellen ist.

## Langfristige Auswirkungen der Corona-Krise

Nach Abflauen der Krise könnte es zu einem grundsätzlichen Umdenken bei vielen Unternehmen über die Grenzen der Globalisierung kommen. Die von deutschen Unternehmen intensiv genutzten internationalen Wertschöpfungsketten versprechen zwar enorme Kostenvorteile, aber die aktuelle Krise führt auch ihre Fragilität vor Augen. Viele Unternehmen werden versuchen, ihre Wertschöpfungsketten stärker zu diversifizieren. So können sie Ausfälle vermeiden, wenn ein länderspezifischer Schock auftritt. Einige werden auch ganz auf Nummer sicher gehen wollen und ihre Produktion gänzlich zurück nach Europa verlagern. Dabei helfen auch neue Technologien, die ein solches „re-shoring“ ermöglichen. Diesen Prozess gilt es konstruktiv zu begleiten und im Auge zu behalten. Insbesondere im Hinblick darauf, was er für die exportorientierte deutsche Volkswirtschaft bedeutet.

So bestehen durchaus Risiken für eine nachhaltige Störung der weltweiten Arbeitsteilung, wenn es zu Veränderungen in der Raumstruktur des Produzierens, zu einer Erhöhung der Fertigungstiefe und einer Neubewertung der Lagerhaltung kommen sollte. Das kann die Investitionsdynamik verändern und besondere Anforderungen an die öffentlichen Infrastrukturen stellen. Zu bedenken ist dabei auch, dass sich die Risikokulisse der Globalisierung bei einer ohnehin verlangsamten Dynamik des Welthandels in den vergangenen fünf Jahren aus mehreren Gründen verändert hat: Wahrnehmung des Epidemierisikos, „Global Power Competition“ und Veränderung der geopolitischen Rahmung der weltwirtschaftlichen Arbeitsteilung sowie Klimarisiken und Migration. In diesem Kontext kann die Corona-Krise als Anlass dienen, die multilaterale Ordnung weiterzuentwickeln. Dies sind allerdings in der Zukunft liegende Herausforderungen. Priorität haben jetzt die kurzfristigen Maßnahmen zur Eindämmung der Verbreitung des Coronavirus und zur Abfederung seiner wirtschaftlichen Auswirkungen.

## References

[CR1] Boone L, Weder di Mauro B, Baldwin R (2020). Tackling the fallout from COVID-19. Economics in the Time of Covid-19.

[CR2] Bundesministerium der Finanzen (2020). Kampf gegen Corona: Größtes Hilfspaket in der Geschichte Deutschlands.

[CR3] Gaspar V, Mauro P (2020). Fiscal Policies to Protect People During the Coronavirus Outbreak. IMFBlog.

[CR4] McKibbin W, Fernando R (2020). The Global Macroeconomic Impacts of COVID-19: Seven Scenarios. CAMA Working paper.

[CR5] World Health Organization (2020). Report of the WHO-China Joint Mission on Coronavirus Disease 2019 (COVID-19).

[CR6] Wren-Lewis S (2020). The economic effects of a pandemic. mainly macro Blog.

